# Reporting verbs across genre sets and research paradigms: a corpus-based analysis of denotation and evaluation in applied linguistics research articles

**DOI:** 10.3389/fpsyg.2026.1799996

**Published:** 2026-04-02

**Authors:** Liming Deng, Xinyue Li

**Affiliations:** 1 English Department, School of Foreign Languages and Literature, Wuhan University, Wuhan, China; 2 Research Institute of Foreign Languages, Wuhan University, Wuhan, China

**Keywords:** denotation, evaluation, genre sets, reporting verbs, research paradigm

## Abstract

**Introduction:**

Reporting verbs are a central linguistic resource that enable writers to represent prior research while negotiating authorial stance with professional community members. While previous studies have documented variation in reporting verb use from various perspectives, less attention has been paid to how its use is shaped by genre sets and research paradigms.

**Methods:**

Drawing on a corpus of 160 research articles in Applied Linguistics, this study investigates the use of reporting verbs across genre sets (pre-methods sections vs. post-methods sections) and research paradigms (quantitative vs. qualitative), with special attention given to their denotations and evaluations.

**Results:**

It is shown that the use of reporting verbs varies across genre sets and research paradigms: pre-methods sections favor verbs denoting research acts and containing neutral evaluation, post-methods sections rely more on discourse-oriented verbs and exhibit a shift toward positive evaluation; quantitative articles tend to employ more research-oriented and neutrally evaluative verbs, while qualitative articles draw more on discourse- and cognition-oriented verbs and adopt more positive evaluative stances.

**Discussion:**

These patterns can be attributed to the combined influence of rhetorical conventions, epistemological orientations, and knowledge-making practices in academic discourse construction. The findings provide pedagogical implications for EAP practitioners in developing novice writers’ awareness of part-genres and research paradigms in their reporting practice when approaching academic writing.

## 1 Introduction

Reporting verbs constitute a key linguistic resource for attributing propositional content to prior sources and negotiating authorial stance in scholarly discourse (Hyland, 2002). Writers typically choose reporting verbs primarily to establish intertextual links of their research with previous studies as the intertextual nature of reporting verbs allows academic writers to indicate their stance toward the reported literature to establish the niche and reinforce the significance of the present research (Swales, 1990).

Despite substantial research on reporting verbs across disciplines (e.g., Cheng et al., 2022; Hyland and Jiang, 2017), writers’ linguistic and/or cultural backgrounds (e.g., Jirapanakorn, 2012; Luzón, 2018; Yilmaz and Erturk, 2017) and English proficiency levels (e.g., Liardét and Black, 2019; Petrić, 2007; Wen and Pramoolsook, 2021), few studies have examined their use across research paradigms, which constitute a fundamental epistemological basis shaping academic writing practice. Moreover, while a number of studies have been carried out within a single part-genre (e.g., Introduction, Cheng et al., 2022; Literature review, Jarkovská and Kučírková, 2020; Conclusion, Deng and He, 2025), or viewing the research articles as a whole (e.g., Hyland and Jiang, 2019; Yasmin et al., 2020), scarce attention has been paid to systematic variation across functional genre sets (e.g., pre-methods vs. post-methods sections).

Previous research has typically focused on the denotation and evaluation of reporting verbs, viewing these two functions as central to knowledge representation and academic interaction in reporting practice (e.g., Cheng et al., 2022; Deng and He, 2025; Hyland, 1999, 2002; Thompson and Ye, 1991). Denotation reflects the type of activities attributed to cited sources and indicates how knowledge is constructed and represented. Evaluation, on the other hand, reveals the authorial stance toward prior studies in response to the communicative demands under certain contexts. These two prominent analytical foci also highlight the dual function of reporting verbs, in which source attribution and authorial evaluation operate simultaneously. They also enable scholars to examine how the linguistic choices align with rhetorical functions and disciplinary knowledge construction. Accordingly, the present study investigates the denotation and evaluation of reporting verbs in Applied Linguistics research articles from cross-part-generic and cross-paradigmatic perspectives, so as to gain insights into the use of reporting verbs across rhetorical units and paradigmatic traditions. The analysis is theoretically informed by Bhatia’s Critical Genre Analysis (CGA) framework, which conceptualizes academic discourse as shaped jointly by the textual choices, generic conventions, professional practices and professional cultures (Bhatia, 2004, 2017). Specifically, the study addresses the following research questions:

*RQ1*: How do genre sets (pre-methods sections vs. post-methods sections) influence the use of reporting verbs in Applied Linguistics research articles?

*RQ2*: How does research paradigm (quantitative vs. qualitative) shape the choice of reporting verbs across Applied Linguistics research articles?

*RQ3*: To what extent do genre sets and research paradigms interact in shaping the denotative and evaluative distributions of reporting verbs in Applied Linguistics research articles?

## 2 Literature review

### 2.1 Reporting verbs

Reporting verbs, such as *analyze, think, argue* and *hypothesize*, are employed to foreground different types of scholarly activities, to convey varied degrees of alignment and evaluation toward previous works, to present arguments that seem to be objective but tactically carry private positions, to situate the writers’ propositions within larger disciplinary communities, and to build authorial images as credible and authoritative (Hyland, 2002; Liardét and Black, 2019; Thompson and Ye, 1991; Zhang, 2022). Extant research has centered on the functions of reporting verbs, particularly their denotation of action types in prior research and their evaluative potential for signaling authorial stance toward cited information (e.g., Charles, 2006a, 2006b; Hyland, 1999, 2002; Thomas and Hawes, 1994; Thompson and Ye, 1991). Such denotative and evaluative functions have made reporting verbs a productive research focus for examining how knowledge claims are constructed and negotiated in disciplinary communities.

Earlier studies have been interested in categorizing and characterizing reporting verbs in academic writing discourse. Thompson and Ye (1991), for example, distinguished reporting verbs from the agent perspective. They referred to the reporting person as the “writer” and the person reported as the “author,” and categorize the evaluative potentials of reporting verbs into author’s stance (i.e., positive, negative, neutral), writer’s stance (i.e., factive, counter-factive, non-factive), and writer’s interpretation (i.e., author’s discourse interpretation, author’s behavior interpretation, status interpretation, non-interpretation). Such a categorization differentiated the positions of the reporting writer and the reported author, making a significant contribution to understanding the evaluation of reporting verbs. However, their categorization involved a rather complex classification that separates evaluative potentials from reporting function while allowing for considerable intercategorial overlapping (Hyland, 2002). By examining 11 research articles on psychosomatic medicine, Thomas and Hawes (1994) further classified reporting verbs according to the acts they represent: real-world or experimental verbs (e.g., *demonstrate, find*), discourse verbs (e.g., *document, state*) and cognition verbs (e.g., *believe, consider*). They also explored the functions of reporting verbs, suggesting a correlation between the verb choices and the rhetorical functions in the reporting practice: real-world/experimental verbs typically report concrete experimental results or generalized empirical conclusions, discourse verbs represent either generalized conclusions or specific research findings, and cognition verbs convey the consensus views of scientific community. Hyland (1999, 2002) expanded previous categorizations by investigating RAs as a whole from hard and soft disciplines to examine reporting verbs from the denotative and evaluative perspective. To him, reporting verbs are classified into research acts verbs representing experimental activities or procedures, discourse acts concerning verbal expression and cognition acts verbs delineating researchers’ mental processes. Reporting verbs can be active verbs indicating the writer’s acceptance of the author’s claims (e.g., *confirm, demonstrate, establish*), non-factive verbs acknowledging no clear attitudinal signal as to their reliability (e.g., *find, observe, identify*) and counter-factive verbs indicating the authors’ judgments as false or incorrect (e.g., *fail, ignore, smisunderstand*). Following Francis et al.’s (1996) grammar patterns of verbs, Charles (2006a, 2006b) examined the reporting verbs according to their semantic functions and categorized them into four semantic groups, namely, ARGUE, FIND, SHOW, and THINK. Hyland and Jiang (2017) further classified the evaluative meanings encoded in reporting verbs, which express different levels of the writer’s judgment or attitude toward the cited research, into positive, negative, and neutral. Taken together, this body of work has established reporting verbs as both ideational and interpersonal resources in academic writing.

Building on these analytical foundations, substantial studies have been carried out to explore the use of reporting verbs across disciplinary contexts (e.g., Cheng et al., 2022; Hyland and Jiang, 2017). Cross-disciplinary research has consistently explored how writers from different disciplinary communities employ reporting verbs to align with the modes of disciplinary knowledge construction, while establishing authorial stance that “reflects the ideology and epistemology of their respective disciplines” (Charles, 2006a, 2006b, p. 493; Cheng et al., 2022; Diani, 2009; Hyland, 1999, 2002; Hyland and Jiang, 2017; Praminatih, 2023). For example, Hyland (1999) explored the citation practices in articles from eight disciplines located in the soft and hard categorization, and discovered significant soft-hard divergence in that the humanities and social sciences disciplines exhibit more frequent use of citations and a preference for reporting verbs pointing to discourse activity, while the sciences and engineering disciplines demonstrate a reliance on reporting verbs that denote research and real-world activities. Charles (2006b) compared MA theses in the field of Politics and Materials science to observe that those in the ARGUE group were the most frequently used reporting verbs in both disciplines, and the reporting verbs in the FIND and SHOW groups appeared with nearly the same frequency in Materials science. More recently, Hyland and Jiang (2017) studied the development of citation practice in journal articles from applied linguistics, biology, engineering and sociology. They observed an increasing use of reporting verbs in applied linguistics and engineering and a decrease in biology and sociology, along with a preference for discourse-oriented verbs in soft disciplines and a favor for research-oriented verbs in hard disciplines. Cheng et al.’s (2022) cross-disciplinary investigation of research article Introductions resonated with Hyland and Jiang’s (2017) findings in that Introductions from applied linguistics articles (i.e., the soft discipline) accounted for a larger proportion of discourse act verbs than the ones in radiology and psychology (i.e., hard disciplines). Apart from comparisons between hard and soft disciplines, studies also discovered variations across sub-categories within the broad hard-soft spectrum, indicating that reporting verbs usage is not only shaped by the hard-soft disciplinary dichotomy, but also influenced by domain-specific professional practices. For example, Diani (2009) investigated reporting verbs use of book review articles of Linguistics, Economy and History, all of which are categorized into soft discipline. It was found that writers from Economy and History tend to present their stance in a rather careful way while those from Linguistics are more direct and polemic. Collectively, these findings suggest a close link between reporting verbs usage and disciplinary knowledge-making, indicating that the denotative and evaluative orientations of reporting verbs are shaped by disciplinary discursive contexts.

Another related while relatively less explored strand of research is the use of reporting verbs across sections of academic writing (e.g., Gao et al., 2025; Olmos-Lopez, 2021; Zhang, 2022). Olmos-Lopez (2021) discovered significant cross-part-generic differences in the use of reporting verbs by analyzing undergraduate theses written by non-native speakers in the field of English language teaching. To be specific, the Literature Review section encompasses the highest frequency of reporting verb use, with discourse act verbs playing the major role in fulfilling the section’s core function of synthesizing previous research. Reporting verbs in the Introduction and Conclusion chapters are used to outline the research background and suggest future directions, respectively. The Results chapter and Methodology chapter, however, have relatively low frequencies of reporting verb use. Similarly, Zhang (2022) examined the use of reporting verbs in Social Sciences articles across the IMRD structure. Cross-part-generic variations were also identified: the Introduction and Discussion sections were the main areas where reporting verbs are distributed, while the Methods and Results sections employed limited amounts of reporting verbs. Additionally, research verbs are predominant in the Introduction and Method sections, representing the procedures and findings of reported work, whereas discourse verbs are preferred in the Result and Discussion sections, emphasizing the viewpoints and arguments of cited literature. Such differences in linguistic choice of reporting verbs are thought to be consistent with the rhetorical functions of each part-genre.

Currently, research on cross-part-genre differences remains relatively limited. Meanwhile, the macro-structure of research articles is far from uniform, and the communicative functions of adjacent sections often overlap, interrelate, or recur (Moreno and Swales, 2018; Samraj, 2005; Yang and Allison, 2003), which poses methodological challenges to section-based data selection and corpus compilation. In view of this, this study situates the comparison of cross-part-generic variations within a broader functional category to regard the pre-methods sections (the Introduction and the Literature Review) and the post-methods sections (the Results, Discussion, and/or closing sections) as two distinct genre sets for analysis. From a genre-analytical perspective, grouping sections into functional genre sets is grounded in the notion that genres rarely operate in isolation but form interrelated components that jointly realize communicative functions (Bazerman, 1994; Devitt, 1991; Samraj, 2005). Accodingly, treating pre-methods and post-methods as coherent genre sets addresses the methodological concern of vague boundaries between generic sections, while also providing reliable units of analysis for examining how reporting verb functions in accordance with the shifting rhetorical functions of different sections in research articles. Therefore, the first objective of our study is to explore the use of reporting verbs across the two genre sets in order to shed light on how the rhetorical functions of different sections influence specific linguistic choice for reporting verbs.

### 2.2 Research paradigms

Paradigm is “a basic set of beliefs that guide action” (Guba, 1990, p.183). In the present study, we refer to paradigm as the approaches to empirical research that are guided by a set of ontological, epistemological and methodological underpinnings. Quantitative and qualitative research are seen as two major approaches that dominate social sciences research (Cohen et al., 2011; Dörnyei, 2007). While quantitative research relies on statistical data to explain social phenomenon and human problem, qualitative research ponders on people, cases, phenomena and social situations in their natural settings to reveal meanings in descriptive terms (Yilmaz, 2013). Different paradigmatic conventions are thought to shape the rhetorical and linguistic choice by which empirical research is presented. Consequently, an increasing number of studies have explored the paradigmatic influence on various discursive resources that characterize academic writing, such as lexical bundles (e.g., Candarli and Jones, 2019; Cao, 2021), metadiscourse (e.g., Cao and Hu, 2014; Hu and Cao, 2015), surprise markers (e.g., Chen and Hu, 2020), and citation practice (e.g., Arizavi and Choubaz, 2021; Gao et al., 2025), and theory use (Gao et al., 2022, 2023).

There is a strand of citation research that has touched on reporting verbs from a cross-paradigmatic perspective (Arizavi and Choubaz, 2021; Chan and Kwan, 2024; Gao et al., 2025; Olmos-Lopez, 2021). For example, Arizavi and Choubaz (2021) analyzed the Introduction sections of Linguistic articles adopting qualitative, mixed and quantitative designs to identify the differences in citation forms and use of reporting verbs. Specifically, quantitative studies were found to employ a larger amount of verb phrases than the quantitative and mixed-method ones. Olmos-Lopez (2021) further observed that the mixed-methods and quantitative approaches tend to employ reporting verbs across all sections of the writing, while qualitative theses use reporting verbs primarily in the earlier theoretical sections. Chan and Kwan (2024) conducted a semantic analysis of citations in Literature Reviews of Information Systems research articles to explore paradigm difference between design science research (DSR) and interpretivist research (IR) traditions. They noted that DSR Literature Reviews prioritize prescriptive propositions, design artifacts, and methodological citations, while IR Literature Reviews emphasize theoretical terminologies, definitions, and explanatory propositions. Gao et al. (2025) investigated the rhetorical functions and linguistic structures of citation practices in post-methods sections of quantitative and qualitative research articles in second language learning and teaching. They revealed that quantitative articles prefer citations that explain results and address limitations, whereas qualitative articles prioritize those recounting and interpreting results. Structurally, quantitative studies favor integral citations without quotations and non-integral citations with introductory phrases, while qualitative studies rely more on non-integral regular citations. These studies have identified cross-paradigmatic differences in citations, providing valuable insights into how distinct epistemological positions within certain research paradigm influence writers’ knowledge construction and epistemological stance-taking in citation practice. However, those studies remain limited in two aspects. First, reporting verbs are often regarded as secondary components of citation research, rather than independent linguistic resources that represent prior research while indicating authorial evaluation. Second, most cross-paradigmatic studies often focus on single sections or the article as a whole, leaving it unclear how paradigm effects interact with the functional variation across research article sub-genres. Therefore, the second objective of our study is to probe into how reporting verbs are used across different research paradigms so as to investigate how the epistemological underpinnings and inquiry conventions of different paradigms shape writers’ reporting verbs choice across genre sets.

### 2.3 The present study

Taken together, prior research has demonstrated that reporting verb use varies across disciplinary contexts and rhetorical sections, yet salient gaps remain to be further explored. First, we still lack a systematic understanding of how the use of reporting verbs varies across functional genre sets to align with the rhetorical functions of different sections. Second, while paradigmatic differences in citation practices have been observed, the role of reporting verbs as micro-level linguistic resources that represent prior work and encode authorial stance remains insufficiently explored. Third, limited attention has been paid to the combined influence of generic and paradigmatic factors, leaving it unclear how these two aspects jointly shape the use of reporting verbs in academic discourse.

The present study addresses these gaps through a corpus-based investigation of reporting verbs across pre-methods and post-methods sections in quantitative and qualitative research articles, focusing on how they denote different types of reported activities and encode evaluative stance toward cited works. The analysis is further informed by Bhatia’s CGA framework, which views genre as a multidimensional construct and posits that the analysis of academic genre is not merely a matter of textual description but also involves explanation of the professional practices and disciplinary cultures (Bhatia, 2004, 2017). Within this framework, variations in reporting verb use are not simply interpreted as lexico-grammatical choices, but also as manifestations of rhetorical functions, epistemic orientations and knowledge-making practices in academic discourse. Accordingly, examining reporting verbs across genre sets and paradigms enables us to link the micro-level linguistic variations with macro-level professional practices.

## 3 Methodology

### 3.1 The corpus

To address the research questions, a total of 160 quantitative and qualitative research articles in the field of applied linguistics were collected. The decision to choose the single discipline of applied linguistics was motivated by two reasons: on the one hand, focusing on a single discipline helps to minimize disciplinary variation; on the other, empirical research articles in this discipline includes a substantial number of both qualitative and quantitative research, yielding data availability for the present research. To ensure the comparability of the data, a set of criteria for data selection was established:

1. *Journal selection*: six prestigious peer-reviewed English-medium journals were targeted with reference to the five-year impact factors (ISI Web of Science, 2023), disciplinary expert opinions and previous research (e.g., Hu and Cao, 2015; Hyland and Jiang, 2017), namely, *Applied Linguistics, Computer Assisted Language Learning, Journal of English for Academic Purposes, ReCALL, Language Teaching Research, and System.*
2. *Timeframe*: empirsical articles published between 2018 and 2022 were included, with 32 articles selected from each publication year;
3. *Text type*: the empirical articles should be well-organized in terms of structures and rhetorical functions, encompassing complete pre-methods and post-methods sections, such as IMRD, ILMRDC, IMRDC, IM[RD]C, etc. (see for the structural patterns in empirical RAs).
4. *Research paradigm*: only articles adopting pure qualitative or pure quantitative approaches were extracted following previous characterizations and practices (e.g., Creswell, 2009; Dörnyei, 2007; Farsani et al., 2021). Articles involving the “tallying, manipulation, or systematic aggregation of quantities of data” (Henning, 1986, p. 702) are recognized as quantitative studies, whereas those employing methods like case study, ethnography and phenomenology are classified into qualitative studies. Regarding articles involving corpus-based analysis and discourse analysis, we adopted Gollin-Kies’ (2014) approach to classify numerically oriented research as quantitative and interpretive research as qualitative, which is also consistent with the classification criteria described in Gao et al. (2025).
5. *Sampling procedure*: after establishing the target journals, the timeframe and annual sample size, and the article selection criteria, random sampling was conducted to obtain a dataset of 160 articles, comprising 80 quantitative and 80 qualitative ones.

The compilation of the corpus involved manual segmentation of texts into pre-methods section (Introduction and Literature review) and post-methods section (Results, Discussion, and closing sections of Conclusion, Implication, Limitation, etc.) and removal of irrelevant part-genres and paratexts (e.g., References, tables, figures, journal tags, page numbers, etc.). In sum, four sub-corpora were built and labeled by section (pre-methods and post-methods) and paradigm (quantitative and qualitative). Table 1 provides the descriptive information of the corpus.

**Table 1:** Descriptive statistics for the corpus.

Research paradigms	No. of articles	Pre-methods sections		Post-methods sections		Total
		Word counts	Mean	Word counts	Mean	
Quantitative articles	80	200,087	2,501	246,252	3,078	446,339
Qualitative articles	80	154,864	1936	287,899	3,599	442,763
Total	160	354,951		534,151		889,102

### 3.2 Coding procedure

Drawing on the taxonomies of previous studies (Hyland, 1999, 2002; Hyland and Jiang, 2017; Thompson and Ye, 1991) and considering the research objectives of the present study, this study analyzed reporting verbs for their denotations, which represent different types of activities (research acts, discourse acts, and cognition acts), and their evaluations, which signal writers’ stance toward cited research (positive, neutral, and negative). Table 2 presents the categorizations with examples (see Table 2).

**Table 2:** Analytical aspects and examples of reporting verbs.

Aspect	Category	Example
Denotation	Research acts	*analyze, demonstrate, find, show, observe*
	Discourse acts	*argue, discuss, explain, suggest, report*
	Cognition acts	*agree, believe, hold, suppose, understand*
Evaluation	Positive	*acknowledge, confirm, point out, show, solve*
	Neutral	*anticipate, find, identify, observe, reflect*
	Negative	*attack, challenge, critique, deny, ignore*

Using UAM Corpus Tool (version 3.3x, O’Donnell, 2019), a free computer program that allows for coding texts at multiple levels, we manually annotated all the corpus data. We started annotation by identifying the reporting structures wherein reference was made in canonical citation form (Swales, 1990): (1) the author’s name followed by a date, either integral (see Example 1) or non-integral (Example 2); (2) the Latinate references to other citations (Example 3). In line with Hyland’s (2002) identification of reporting verbs, cases of self-citation were left out to avoid conflating evaluative attribution to outside voice with authorial self-representation, thus ensuring the validity of evaluation analysis. Forms such as a number in squared brackets and superscript references were not examined. Verbs involved in a reporting structure would be identified as reporting verbs and tagged with their exact denotation and evaluation.

Example (1): Leonardo (2009) demonstrate how colorblind discourse has been obfuscated rather than halted racialization process (Pre_Qual_09).

Example (2): Decades of research into L2 communicative competence highlights the complex mix of factors involved in good interactional abilities (Lennon, 1990; Ortega, 2011; Savignon, 2007) (Pre_Quan_01).

Example (3): In their study, Chen and Sun (ibid.) compared the impact of three multimedia materials (Pre_Quan_16).

Following the above analytical framework, one coder (one of the authors) independently analyzed all the tagged reporting verbs for their denotation and evaluation. To ensure the inter-coder reliability, another coder (a doctoral student in applied linguistics) was invited to annotate 15% of the text in each sub-corpora. Inter-coder reliability was calculated through Cohen’s Kappa in SPSS 25.0 for each analytical dimension. The values for inter-rater reliability are 0.85 for denotation and 0.91 for evaluation, indicating high-level agreements between the two coders.

### 3.3 Data analysis

We adopted both statistical analysis (using SPSS 26) and textual analysis on the coded data. To examine the use of reporting verbs across genre sets and paradigms (RQ1-RQ2), we carried out a series of Chi-square tests of Independence on the raw frequency data. Bonferroni correction was applied due to four separate chi-square tests of each analytical aspects (namely, denotation and evaluation), with the alpha value set at 0.0125. Cramer’s V values were calculated as effect size indicators, with values between 0.06 and 0.17 indicating small effects, 0.18–0.30 denoting medium effects, and values >0.30 representing large effects. Standardized residual analyses were conducted as a post-hoc test to identify the specific categories driving the statistically significant results. To examine the interaction between genre sets and research paradigms (RQ3), we conducted log-linear analyses on the distributions of reporting verbs across genre sets and paradigms within each analytical dimension. Additionally, salient instances were extracted to complement the quantitative data by exemplifying how reporting verbs are utilized in different genre sets and paradigms.

## 4 Results

Table 3 summarizes the descriptive statistics for different aspects of reporting verbs use across genre sets and paradigms. Table 4 presents statistical comparisons of reporting verbs across genre sets and research paradigms, including Chi-squares tests of distributional differences and log-linear analyses examining interaction effects. In the following sections, we present the statistical results with representative examples from each aspect involved in our analytical framework.

**Table 3:** Descriptive statistics for reporting verbs across genre sets and research paradigms.

Aspect	Category	QUAN						QUAL					
		Pre-methods sections			Post-methods sections			Pre-methods sections			Post-methods sections		
		Raw	%	Per 1,000	Raw	%	Per 1,000	Raw	%	Per 1,000	Raw	%	Per 1,000
Denotation	Research	1,396	54.47%	6.98	281	47.63%	1.17	850	45.19%	5.49	153	29.77%	0.53
	Discourse	998	38.94%	4.99	270	45.76%	1.12	889	47.26%	5.74	304	59.14%	1.06
	Cognition	169	6.59%	0.84	39	6.61%	0.16	142	7.55%	0.92	57	11.09%	0.2
Evaluation	Positive	998	38.93%	4.99	270	45.76%	1.12	957	50.88%	6.18	273	53.11%	0.95
	Neutral	1,511	58.99%	7.55	307	52.03%	1.28	878	46.68%	5.67	233	45.33%	0.81
	Negative	54	2.10%	0.27	13	2.20%	0.05	46	2.44%	0.3	8	1.56%	0.03

**Table 4:** Statistical comparisons of reporting verbs across genre sets and research paradigms.

Aspect	Comparison		*χ* ^2^	df	*N*	*p*	Cramer’s V
Denotation	Pre vs. Post	QUAN	9.77	2	3,153	0.008	0.056
		QUAL	40.46	2	2,395	<0.001	0.130
	QUAN vs. QUAL	Pre	37.59	2	4,544	<0.001	0.091
		Post	38.09	2	1,104	<0.001	0.185
	Genre sets × Paradigm interaction		3.23	1		0.071	
Evaluation	Pre vs. Post	QUAN	9.56	2	3,153	0.008	0.056
		QUAL	1.97	2	2,395	0.374	0.028
	QUAN vs. QUAL	Pre	66.12	2	4,544	<0.001	0.130
		Post	6.15	2	1,104	0.046	0.074
	Genre sets × Paradigm interaction		4.89	1		0.027	

### 4.1 The use of reporting verbs in pre- and post-methods sections of research articles

The first research question aims to explore the influence of genre sets (namely, pre-methods sections and post-methods sections) on the denotation and evaluation of reporting verbs in research articles. As is shown in Table 4, for quantitative articles, the chi-square tests revealed significant influences of genre sets on the distribution of denotations (*χ*^2^ = 9.77, *p =* 0.008, Cramer’s V = 0.056) and evaluations (*χ*^2^ = 9.56, *p =* 0.008, Cramer’s V = 0.056), although both effects were small. Residual analysis demonstrated that the differences were mainly driven by research verbs and neutrally evaluative verbs in pre-methods sections, and discourse verbs and positive evaluative verbs in post-methods sections. The results suggested that in terms of denotative orientation, research-oriented verbs occurred more often in pre-methods sections, while discourse-oriented verbs were more common in the post-methods sections. Regarding evaluative potential, pre-methods sections relied more on neutral verbs, whereas post-methods sections showed a modest shift toward positive evaluation.

In qualitative articles, a significant influence of genre sets on denotative function distribution was detected (*χ*^2^ = 40.46, *p <* 0.001, Cramer’s V = 0.130), reaching a modest magnitude. However, no significant difference was found between pre- and post-method sections regarding the evaluation of reporting verbs (*χ*^2^ = 1.97, *p =* 0.374, Cramer’s V = 0.028). Residual analysis showed that variation across genre sets was primarily driven by research-oriented verbs in pre-methods sections and discourse-oriented verbs in post-methods sections. Our findings indicated that pre-methods sections demonstrated a preference for research research-oriented verbs, whereas the post-methods sections exhibited a showed declining use of research verbs and increased reliance on discourse-oriented verbs and cognition-oriented verbs. The proportions of positive, neutral, and negative reporting verbs remained relatively stable across sections, indicating that genre sets exerted no statistically significant influence on the evaluation of reporting verbs in qualitative research writing.

Collectively, genre sets have a significant influence on the distribution of reporting verbs with respect to their denotative orientations and evaluative potentials, albeit to varying degrees across research paradigms. These quantitative tendencies can also be exemplified by instances in corpus. As shown in Example 4, the use of a research verb indicates that the writer is more inclined to refer to the research methodology of the reported work in pre-methods sections. Example 5 demonstrates how writers employ discourse-oriented verbs to underline previous findings and situate the present study within ongoing scholarly discussions. Example 6 illustrates how writers incorporate varied degrees of evaluation to position cited claims in post-methods sections. The verb “*argued*” functions as a marker of neutral evaluation, simply reporting prior work stance without explicit endorsement. Whereas “*asserting*” represents an instance of positive evaluation, emphasizing the strength of the cited claim and implicitly aligning the current research with the core findings.

Example 4: A growing number of studies have examined how technology can be utilized to promote teachers’ RP and professional learning in the past years (Cherrington and Loveridge, 2014; Lee, 2010) (Intro_Qual_01).

Example 5: The results were aligned with Lai et al.’s (2012) findings, which highlighted facilitating conditions as an important force of technology acceptance (RD_Qual_30).

Example 6: Schmidt (2001) argued that the learning of any feature of the second language cannot occur without noticing, asserting that “while there is subliminal perception, there is no subliminal learning” (p. 26) (RD_Quan_09).

### 4.2 The use of reporting verbs in quantitative and qualitative research articles

The second research question attempts to address how research paradigm (quantitative research articles vs. qualitative research articles) influences the use of reporting verbs regarding their denotative and evaluative functions in research articles. In the pre-methods sections, the chi-square tests showed significant differences between quantitative and qualitative research articles in terms of the denotation (*χ*^2^ = 37.59, *p <* 0.001, Cramer’s V = 0.091) and evaluation (*χ*^2^ = 66.12, *p <* 0.001, Cramer’s V = 0.130). Residual analysis indicated that the differences stemmed from research-oriented verbs and neutrally evaluative verbs in quantitative writing and discourse verbs and positive evaluation in qualitative writing. Evidently, for the denotation of reporting verbs, research-oriented verbs were more commonly used in quantitative articles, while a more balanced use of discourse-oriented verbs and research-oriented verbs in qualitative articles occurred. With regard to the evaluative potentials, quantitative articles relied more on neutral evaluation, whereas qualitative articles displayed a preference for positive evaluation compared to quantitative articles, with negative evaluation playing a limited role.

In the post-methods sections, paradigmatic differences remained significant in the denotation of reporting verbs (*χ*^2^ = 38.09, *p <* 0.001, Cramer’s V = 0.185), and reached statistical significance for evaluation in terms of evaluation (*χ*^2^ = 6.15, *p =* 0.046, Cramer’s V = 0.074), though with a small effect size. Residual analysis showed that differences in denotation was driven merely by research verbs in quantitative research and discourse verbs in qualitative writing, with the evaluative dimension making no contribution to the marginal difference. The analyses revealed that, for the denotative orientations, quantitative articles demonstrated a reliance on verbs denoting research acts, whereas qualitative ones exhibited a preference for discourse-oriented verbs and cognition-oriented ones. Concerning the evaluations, the paradigmatic comparison detected a limited variation, with quantitative articles demonstrated a nuanced reliance on neutrally evaluative verbs, while qualitative articles continued to choose positive evaluations slightly more.

Taken together, paradigmatic variation is consistently evident regarding denotation while less consistent in evaluation, being more pronounced in pre-methods sections than in post-methods sections. For instance, the research-oriented verb in Example 7 explains how the cited work was carried out to highlight its methodological stringency. Example 8 and Example 9 demonstrate how qualitative articles encode their engagement with the interpretive and conceptual dimensions of prior scholarship through discursive reasoning and mental processing. Example 10 exemplifies the use of neutral evaluation to withhold judgments in quantitative inquiry. Example 11 illustrates how qualitative researchers employing positive evaluation to associate themselves with prior knowledge while implicitly supporting the present findings.

Example 7: Tsai (2019) evaluated Chinese students’ written texts using two types of online assessments (LR_Quan_33).

Example 8: This might be relevant to the unique features of Chinese characters, as discussed in previous studies (Lee and Smith, 2011; Moloney and Xu, 2015) (RD_Qual_70).

Example 9: The negotiation work undertaken while following these steps has been considered valuable for facilitating L2 acquisition (Long, 1996), which can also be extended to L2 pedagogy through the implementation of TBLT procedures (LR_Qual_50).

Example 10: This coverage ensures an accurate and valid estimate of students’ overall level of FL speaking anxiety, which has been found to be lacking in previous administrations of the FLCAS (Panayides and Walker, 2013; Walker and Panayides, 2014) (RD_Quan_56).

Example 11: Ruohotie-Lyhty and Moate (2016) held that teacher agency is a teacher’s “capacity to influence, make choices, and take stances in ways that affect one’s work” (p. 319).(RD_Qual_22).

### 4.3 The use of reporting verbs across genre sets and research paradigms

The third research question seeks to examine how genre set (pre-methods sections vs. post-methods sections) and research paradigm (quantitative vs. qualitative research articles) jointly influence the use of reporting verbs in terms of their denotative and evaluative functions in research articles. Log-linear analyses were conducted to examine whether genre sets and research paradigms interacted in shaping the distributions of reporting verbs. For denotation, genre sets and research paradigms showed no statistically significant interaction (*χ*^2^ = 3.23, *p =* 0.071), although there was a tendency that the shifts of denotative orientations across sections were more pronounced in qualitative articles. This indicates that the denotative distribution of reporting verbs across genre sets was broadly comparable across paradigms. For evaluation, the interaction was significant (*χ*^2^ = 4.89, *p =* 0.027), suggesting that evaluative distributions are more markedly influenced by the joint influence of rhetorical sections and research paradigms. In quantitative articles, reporting verbs exhibited a clear increase in positive evaluation from the pre-methods sections to post-methods sections. In qualitative articles, by contrast, evaluation of reporting verbs remained relatively stable across genre sets. Such patterns suggest that authorial stance encoded in reporting verbs is more consistent throughout qualitative articles, whereas it varies more substantially across genre sets in quantitative writing.

## 5 Discussion

### 5.1 Generic influence on the use of reporting verbs

Variations in the choice of reporting verbs across genre sets could be attributed to the influence of different rhetorical functions of pre- and post-methods sections of research articles. The pre-methods sections, including the Introduction and the Literature Review, function to situate the current study within the existing body of scholarship by establishing the research territory, identifying a niche, and justifying the present inquiry (Chen and Li, 2019; Kwan et al., 2012; Swales, 1990; Zhang, 2022). Consistent with such generic demands, reporting verbs in the pre-methods sections tend to foreground the research-related activities of the cited studies, as reflected in the predominance of research-oriented verbs in these sections (Cheng et al., 2022; Zhang, 2022). By emphasizing empirical actions and methodological procedures, writers are better able to highlight the methodological robustness and evidential value of prior works, thereby creating a research space for the current study. Regarding the evaluation of reporting verbs, quantitative articles relies mostly on neutral verbs, while qualitative studies employ more positive and neutral verbs, reflecting a more endorsing stance toward the cited works in the early stages of qualitative writing. This pattern fulfills the dual rhetorical function of reporting: presenting prior research as a credible and coherent knowledge base (Liardét and Black, 2019) and justifying the research gap (Deng et al., 2024b). Through such denotative and evaluative choices, writers seek to both map out the scholarly network of the field and position themselves strategically within it, so as to reinforce the rationale of the research before new knowledge is introduced.

The post-methods sections, typically comprising the Results, Discussion, and/or closing sections, function to report and interpret empirical findings and articulate the research contribution and implications (Moreno, 2021; Moreno and Swales, 2018; Deng et al., 2024b; Yang and Allison, 2003). Their communicative functions shifts from justifying the research to interpreting and evaluating research findings as well as highlighting research significance. From the denotative perspective, discourse- and cognition-oriented verbs become more frequent, indicating a growing emphasis on interpreting the findings and aligning them with prior claims (Samraj, 2005; Deng et al., 2025; Zhang, 2022). With regard to evaluation, these sections display a shift from neutral attitude to positive stance, suggesting that writers are more inclined to endorse prior works when interpreting findings and articulating contribution. To sum up, the increase use of discourse- and cognition-oriented denotation and positive evaluation reveals that the post-methods sections serve as a space where reporting verbs function less to establish the scholarly territory but more to explain, compare, and integrate new knowledge within the academic community (Deng et al., 2024a; Deng et al., 2025).

This cross-sectional variation is consistent with prior findings that citation functions differ across pre- and post-methods sections (e.g., Gao et al., 2025), and our findings also suggest that such functional shifts are reflected at the linguistic level.

### 5.2 Paradigmatic influence on the use of reporting verbs

The observed differences in the use of reporting verbs between quantitative and qualitative research articles can be interpreted from two interrelated perspectives: the epistemological underpinnings of the two paradigms and the knowledge-making practices through which each paradigm constructs and legitimates claims.

At the epistemological level, quantitative research is typically grounded in a postpositivist epistemology that emphasizes the general law of cause-and-effect, and conceptualizes knowledge as deriving from observed phenomena, empirical procedures and rationale thinking (Phillips and Burbules, 2000; Creswell, 2009). Correspondingly, quantitative articles prefer to construe prior studies as research activity and to employ neutral evaluation to maintain impersonal and cautious stance (Arizavi and Choubaz, 2021; Gao et al., 2025). Such denotative and evaluative choices reflect the norms of objectivity, methodological rigor, and epistemic caution that characterize quantitative knowledge production (Cohen et al., 2011; Cao and Hu, 2014). Qualitative research, by contrast, is informed by a social constructivist worldview, viewing knowledge as emerging from human experience, subjective interpretation and social interaction (Creswell, 2009; Dörnyei, 2007). It therefore prefer discourse- and cognition-oriented verbs and more positive evaluation, especially in the pre-methods sections. These functional choices enable writers to engage explicitly with prior arguments and establish intertextual links with existing research.

At the level of professional practice, the quantitative-qualitative divergence in functional distribution of reporting verbs could be explained by the distinct knowledge-making performance of the two paradigms (Cao and Hu, 2014; Gao et al., 2022; Hu and Cao, 2015; Maton, 2000). Aligning with Maton (2000) conceptualization of “knowledge-knower mode,” quantitative inquiry involves a deductive pattern that legitimate knowledge through methodological rigor and empirical evidence (Dörnyei, 2007), aligns it more closely with the knowledge mode. Consistent with prior research on cross-paradigmatic citation practices (Arizavi and Choubaz, 2021; Gao et al., 2025), our findings further show that quantitative research favors research-oriented verbs to to integrate prior work as procedural and evidential foundations to legitimate the current study, and neutral evaluation to withhold judgements toward outside voice and position authorial stance as objective. Qualitative inquiry, on the other hand, inductively generates patterns of meaning that arises from human interaction and interpretation of other’s meaning of the world. It leans toward the knower mode, where knowledge is warranted not only by methodological adequacy but also by the researchers’ interpretation (Cao and Hu, 2014; Hu and Cao, 2015; Maton, 2000), and prior research is not merely referred as the background but it is often engaged dialogically as interpretive resource (Arizavi and Choubaz, 2021; Gao et al., 2025). Therefore, qualitative writers use more discourse- and cognition-oriented verbs and more positive evaluation to strategically facilitate alignment with previous viewpoints and engage themselves within the scholarly dialogue. Additionally, the post-methods sections in qualitative articles demonstrate stronger paradigmatic effects on denotation but relatively weaker effects on evaluation. This pattern suggests that although paradigms differ in how writers construe reported activity, authorial evaluation toward prior literature is more strongly regulated by the shared communicative purposes of post-methods sections.

### 5.3 Combined influence of genre sets and research paradigms on the use of reporting verbs

The interaction analysis provides a more integrated account of how genre sets and research paradigms work jointly in shaping the use of reporting verbs. For denotation, the functional distribution of reporting verbs across sections is largely consistent across different paradigms, with no substantial variation between paradigms. This indicates that the influence of genre sets is relatively stable. Both quantitative and qualitative studies rely largely on research-oriented verbs in the pre-method sections, and discourse- and cognition-oriented verbs in the post-methods sections. This pattern corroborates the view that the use of reporting verb typically varies to represent different types of activities and accommodate genre-specific rhetorical functions (e.g., Cheng et al., 2022; Un-udom and Un-udom, 2020; Zhang, 2022). Regarding evaluative potentials of reporting verbs, a clear interactive pattern was discovered. Quantitative articles demonstrate notable differences across pre- and post-methods sections, whereas qualitative articles maintain a relatively stable evaluation in both sections. This indicates that the evaluation encoded in reporting verbs is more strongly associated with the epistemological orientation and knowledge-making mode of the research paradigm.

By jointly modeling genre sets and research paradigms as interacting contextual factors, this study provides a more integrated explanatory account of how reporting verbs are strategically deployed in academic discourse. The observed patterns suggest that variation in reporting verbs emerges from the intersection of genre-specific rhetorical demands and paradigm-situated epistemic orientations and knowledge practices. Collectively, the findings reveal that reporting verbs align with contextualized communicative purposes, encode paradigm-specific epistemic orientations while aligning with the distinct modes of knowledge-making, therefore linking the micro-level linguistic choices with the macro-level professional practice. The findings further develop existing accounts of reporting verb use by foregrounding how rhetorical, epistemic and professional factors jointly shape writers’ reporting practice.

## 6 Conclusion

This study has examined the use of reporting verbs across genre sets (pre-methods sections vs. post-methods sections) and research paradigms (quantitative vs. qualitative), focusing on the denotative orientations and evaluative potentials of reporting verbs. Overall, the findings indicate that genre sets exert a systematic influence on the denotative and evaluative functions of reporting verbs, manifested by the shifts in rhetorical focus from establishing empirical foundations in the pre-methods sections to negotiating new claims within academic community in the post-methods sections. Specifically, pre-methods sections are characterized by a preference for reporting verbs denoting research activities and carry neutral evaluation, whereas post-methods sections make greater use of discourse-oriented verbs and show an increasingly positive stance toward the reported work. Clear paradigmatic differences were also observed, indicating that quantitative and qualitative research mobilize reporting verbs in distinct ways that align with their epistemological orientations and knowledge-making practices. Quantitative articles tend to prefer reporting verbs denoting research acts and display neutral evaluation towards outside knowledge, whereas qualitative articles make more use of discourse- and cognition-oriented verbs and exhibit more positive evaluation, particularly in the pre-methods sections. Notably, the interaction analysis further demonstrates that while the denotative orientations of reporting verbs remain consistent across research paradigms, their evaluative potential varied across paradigms and genre sets.

Taken together, these findings demonstrate that the use of reporting verbs is shaped jointly by genre-based rhetorical conventions and paradigm-specific epistemic foundations and disciplinary practices. The findings also provide empirical evidence for Bhatia’s (2004, 2017) Critical Genre Analysis framework that incorporates textual, generic and professional dimensions of discourse construction. Moving beyond previous studies that view reporting verbs either as isolated lexical choices or as uniform markers of stance, the present study establishes a more integrated account of analysis by linking the linguistic phenomenon with rhetorical, epistemological and professional aspects of academic writing. It situates the scholarship within a territory that takes into account both text-internal (e.g., linguistic choices, generic conventions) and text-external features (e.g., epistemic orientations, professional practice), as strongly advocated by Bhatia (2004, 2017).

The findings of this study have implications for EAP writing and instruction. First, the variation in reporting verbs use across pre-methods and post-methods sections suggests that writing instruction should go beyond presenting reporting verbs as a homogeneous linguistic resource and underscores their genre-specific usage for varied communicative purposes. Students can be guided to recognize the distinct rhetorical functions of RA part-genres and be equipped with appropriate discursive strategies tailored to genre-specific rhetorical needs. Second, the observed paradigmatic differences indicate the necessity to raise learners’ awareness of how quantitative and qualitative research traditions mobilize reporting verbs to construe prior work and signal authorial evaluation. Therefore, explicit instruction that links reporting verb choice to research design, inquiry logic, and knowledge-making mode needs to be adequately implemented within the classroom teaching so as to help novice student writers better align their linguistic choices with disciplinary and professional expectations. Yet the instruction should not be merely limited to pure quantitative or qualitative studies given the growing interest in mixed methods research in the domain of applied linguistics (Farsani et al., 2021). Accordingly, instructors could help raise novice writers’ paradigmatic awareness by illustrating how reporting verb choices align with the quantitative and qualitative components of research designs, thereby offering tentative insights into effective reporting practices within the disciplinary context.

Despite the implications, this study is subject to several limitations. First, it merely focused on linguistics articles with the endeavor to delve into the reporting verb within one single discipline, which might constrain the generalizability of the findings to other disciplines. Given the limit, more disciplines and their sub-fields should be involved to uncover paradigmatic variations on a larger scale. Second, although the division of research articles into genre sets of pre-methods and post-methods sections allows for a functional comparison that accommodates structural variation across journals and articles, this approach may not fully capture the use of reporting verbs within generic units like moves and steps of a certain part-genre. It would be insightful to carry out studies that incorporate reporting verbs into the generic structures to explore how reporting verbs align with the rhetorical functions of certain move-steps so as to bridge the form-function gap on a more fine-grained level. Future research are suggested to integrate reporting verbs analysis with genre-based approaches within a larger disciplinary domain, so that scholars could better understand how writers from diverse disciplinary and professional backgrounds use reporting verbs to position prior research and achieve their communicative purposes.

## Data Availability

The raw data supporting the conclusions of this article will be made available by the authors, without undue reservation.
